# Sobrepeso, obesidade e fatores associados à saúde em afrodescendentes hipertensos residentes em comunidade Quilombola no Brasil

**DOI:** 10.15446/rsap.V27n1.109946

**Published:** 2025-01-01

**Authors:** Helder Caldas Torres, Randson Souza Rosa, Jean Carlos Zambrano Contreras, José de Bessa, Rita Narriman Silva de Oliveira Boery, Isleide Santana Cardoso Santos, Wilkslam Alves de Araújo, Diego Pires Cruz, Edison Vitório de Souza, Ronney Pereira Cabral

**Affiliations:** 1 HC: Enf. Investigador, Curso Enfermagem e Obstetrícia. Universidade Estadual do Sudoeste da Bahia/UESB. Jequié (BA), Brasil. hctorres5854@gmail.com Universidade Estadual do Sudoeste da Bahia Universidade Estadual do Sudoeste da Bahia Jequié BA Brazil; 2 RS: Enf. M. Sc. Ciências da Saúde. Investigador, Programa de Pós-Graduação em Saúde Coletiva da Universidade Estadual de Feira de Santana (UEFS). Feira de Santana (BA), Brasil. enfrandson@gmail.com Universidade Estadual de Feira de Santana Universidade Estadual de Feira de Santana Feira de Santana BA Brazil; 3 JZ: EF. Ph. D. Ciências da Atividade Física e o Esporte. Ph. D. Saúde Coletiva. Investigador, Universidade Estadual de Feira de Santana (UEFS). Feira de Santana (BA), Brasil. zambrano.jeancarlos@gmail.com Universidade Estadual de Feira de Santana Universidade Estadual de Feira de Santana Feira de Santana BA Brazil; 4 JB: MD. Ph. D. Ciências. Docente, Programa de Pós-Graduação em Saúde Coletiva (PPGSC) Universidade Estadual de Feira de Santana (UEFS). Feira de Santana (BA), Brasil. bessa@uefs.br Universidade Estadual de Feira de Santana Universidade Estadual de Feira de Santana Feira de Santana BA Brazil bessa@uefs.br; 5 RN: Enf. Pós-doutora em Bioética. Ph. D. Enfermagem. Docente, Programa de Pós-Graduação em Enfermagem e Saúde (PPGES), Universidade Estadual Sudoeste da Bahia (UESB). Jequié (BA), Brasil. rboery@gmail.com Universidade Estadual do Sudoeste da Bahia Universidade Estadual Sudoeste da Bahia Jequié BA Brazil; 6 IS: Enfe. Ph. D. Ciências da Saúde. Docente, Programa de Pós-Graduação em Enfermagem e Saúde (PPGES), Universidade Estadual do Sudoeste da Bahia (UESB). Jequié (BA), Brasil. isantana@uesb.edu.br Universidade Estadual do Sudoeste da Bahia Universidade Estadual do Sudoeste da Bahia Jequié BA Brazil isantana@uesb.edu.br; 7 WA: Enf. Ph. D. Ciências da Saúde. Investigador, Programa de Pós-Graduação em Enfermagem e Saúde (PPGES), Universidade Estadual do Sudoeste da Bahia (UESB). Jequié (BA), Brasil. wilkslam@hotmail.com Universidade Estadual do Sudoeste da Bahia Universidade Estadual do Sudoeste da Bahia Jequié BA Brazil; 8 DP: Enf. Ph. D. Ciências da Saúde. Investigador, Programa de Pós-Graduação em Enfermagem e Saúde (PPGES), Universidade Estadual do Sudoeste da Bahia (UESB). Jequié (BA), Brasil. diego_pcruz@hotmail.com Universidade Estadual do Sudoeste da Bahia Universidade Estadual do Sudoeste da Bahia Jequié BA Brazil; 9 ES: Enf. Ph. D. Ciências da Saúde. Investigador, Escola de Enfermagem de Ribeirão Preto da Universidade de São Paulo (EERP/USP). São Paulo (SP), Brasil. edison.vitorio@gmail.com Universidade de São Paulo Universidade de São Paulo São Paulo SP Brazil; 10RP: Enf. Ph. D. Ciências da Educação. Docente, Curso de Enfermagem, Universidade Estadual do Sudoeste da Bahia (UESB). Jequié (BA), Brasil. ronney.cabral@yahoo.com.br Universidade Estadual do Sudoeste da Bahia Universidade Estadual do Sudoeste da Bahia Jequié BA Brazil

**Keywords:** Sobrepeso, obesidade, hipertensão, fatores de risco, doenças cardiovasculares, quilombolas, saúde de população negra *(fonte: DeCS, BIREME)*, Overweight, obesity, hypertension, risk factors, cardiovascular diseases, quilombolas, black population health *(source: MeSH, NLM)*, Sobrepeso, obesidad, hipertensión, factores de riesgo, enfermedades cardiovasculares, quilombolas, salud de la población negra *(fuente: DeCS, BIREME)*

## Abstract

**Objetivo:**

Analisar o sobrepeso, obesidade e os fatores associados à saúde em afrodescendentes hipertensos residentes em uma comunidade quilombola.

**Métodos:**

Trata-se de um estudo epidemiológico, censitário, de delineamento transversal e de base comunitária, realizado com 303 residentes de uma comunidade quilombola.

**Resultados:**

Os resultados com os 303 afrodescendentes hipertensos, em sua maioria mulheres, identificou os principais fatores de risco cardiovascular com destaque para a menopausa, histórico familiar de problemas cardiovasculares, sedentarismo e diabetes tipo 2. Observou-se diferenças significativas entre homens e mulheres nos níveis de colesterol total, HDL e LDL. Uma análise de regressão logística indicou que sexo feminino, e altos níveis de colesterol total estavam associados a um índice de massa corporal (IMC) mais elevado. Essas descobertas fornecem insights importantes sobre os fatores de risco cardiovasculares em afrodescendentes hipertensos, destacando a importância da identificação precoce e manejo desses fatores para a prevenção de doenças cardiovasculares nessa população.

**Conclusões:**

O contexto do estudo reforça a necessidade de estratégias preventivas e intervencionistas para combater o sobrepeso e a obesidade nas comunidades quilombolas. O conhecimento dos riscos cardiovasculares e fatores associados é essencial para a implementação de intervenções eficazes e contribui como uma ferramenta para direcionar ações de saúde e políticas sociais nas comunidades quilombolas e em outras populações vulneráveis.

## Abstract (en)

**Objective:**

To analyze overweight, obesity and factors associated with health in hypertensive Afro-descendants living in a quilombola community.

**Methods:**

This is an epidemiological, census, cross-sectional, community-based study carried out with 303 residents of a quilombola community.

**Results:**

The results with 303 hypertensive Afro-descendants, mostly women, identified the main cardiovascular risk factors with emphasis on menopause, family history of cardiovascular problems, sedentary lifestyle and type 2 diabetes. Significant differences were observed between men and women in the levels of total cholesterol, HDL and LDL. Logistic regression analysis indicated that female sex, and high levels of total cholesterol were associated with a higher body mass index (BMI). These findings provide important insights into cardiovascular risk factors in hypertensive African Americans, highlighting the importance of early identification and management of these factors for the prevention of cardiovascular disease in this population.

**Conclusions:**

The context of the study reinforces the need for preventive and interventionist strategies to combat overweight and obesity in quilombola communities. Knowledge of cardiovascular risks and associated factors is essential for the implementation of effective interventions and contributes as a tool to direct health actions and social policies in quilombola communities and other vulnerable populations.

## RESUMEN

**Objetivo:**

Analizar el sobrepeso, la obesidad y los factores asociados a la salud en afrodescendientes hipertensos residentes en una comunidad quilombola.

**Métodos:**

Estudio epidemiológico, censal, transversal, de base comunitaria, realizado con 303 habitantes de una comunidad quilombola.

**Resultados:**

Los resultados, con 303 afrodescendientes hipertensos, en su mayoría mujeres, identificaron los principales factores de riesgo cardiovascular con énfasis en la menopausia, antecedentes familiares de problemas cardiovasculares, sedentarismo y diabetes tipo 2. Se observaron diferencias significativas entre hombres y mujeres en los niveles de colesterol total, HDL y LDL. El análisis de regresión logística indicó que el sexo femenino y niveles altos de colesterol total se asociaron con un mayor índice de masa corporal (IMC). Estos hallazgos brindan información importante sobre los factores de riesgo cardiovascular en los afroamericanos hipertensos, lo que destaca la importancia de la identificación y el manejo tempranos de estos factores para la prevención de la enfermedad cardiovascular en esta población.

**Conclusiones:**

El contexto del estudio refuerza la necesidad de estrategias preventivas e intervencionistas para combatir el sobrepeso y la obesidad en comunidades quilombolas. El conocimiento de los riesgos cardiovasculares y los factores asociados es fundamental para la implementación de intervenciones efectivas y contribuye como herramienta para orientar las acciones de salud y las políticas sociales en las comunidades quilombolas y otras poblaciones vulnerables.

De acordo com a Organização Mundial da Saúde o sobrepeso e a obesidade são definidos como o acúmulo de gordura corporal em uma quantidade que determine prejuízos à saúde. Essa quantidade de gordura corporal pode ser mensurada pelo índice de massa corpórea (IMC) e a OMS afirma que adultos com IMC entre 25 e 29,9 kg/m^2^ são considerados com sobrepeso que é uma característica que já pode acarretar alguns prejuízos a saúde, enquanto pessoas com o índice maior ou igual a 3okg/m2 são considerados indivíduos obesos 1.

Em um estudo com afrodescendentes residentes em comunidade, evidenciou que entre os adultos quilombolas, o sobrepeso e o acúmulo de gordura abdominal foram as alterações nutricionais mais relevantes, especialmente entre as mulheres. Esses problemas estão associados a uma série de fatores investigados, como fatores socio-demográficos (como raça e condição socioeconômica), comportamentais (como hábitos alimentares e atividade física) e de saúde. Ainda no mesmo estudo notou-se a presença do sobrepeso e da obesidade abdominal nessa população, predominantemente de cor/raça preta e homogeneamente pobre, refletindo a complexidade das variáveis que contribuem para esses problemas de saúde 2.

O sobrepeso e a obesidade têm sérias repercussões negativas no processo saúde-doença dos indivíduos. São considerados prováveis preditores de eventos cardiovasculares e fazem parte de um contexto maior de condições crônicas não transmissíveis em saúde pública. Além disso, contribuem para os impactos negativos no sistema de saúde, principalmente na atenção primária à saúde, com foco nas complicações relacionadas às doenças circulatórias. Isso também afeta as internações e os indivíduos que vivem em seu contexto familiar e comunitário 3.

Os residentes em quilombos são um grupo vulnerável para o desenvolvimento de doenças crônicas, principalmente a hipertensão arterial, devido às más condições de saúde que interferem diretamente em seus estilos de vida 4.

Os povos quilombolas representam uma parte da população que historicamente resistiu a um período conturbado de escravidão, onde havia um processo de idealização que a parcela da população afrodescendente era sinônimo de falta de desenvolvimento e de não civilizados, sofrendo assim preconceitos muito fortes 5.

A parcela da população residente em quilombos é formada por moradores de áreas rurais, semiurbanas e, ainda, uma minoria que vive em áreas urbanas. Os quilombolas somam uma parte representativa da população afro-brasileira, porém formam uma parcela que são, de certa forma, esquecidas pela sociedade 6.

A hipertensão é um fator de risco cardiovascular que afeta muito a parte da população de ascendência africana, principalmente as que vivem em quilombos, isso se deve também pelas baixas condições econômicas e desigualdades sociais vividas pelos povos quilombolas, é perceptível que os maiores casos de problemas de saúde são revelados em quantidade superior nessa parcela da população 6.

Pesquisas científicas sobre os indivíduos residentes em quilombos ainda são pouco numerosas e para entender sobre as condições de saúde, sociais e econômicas dessa parcela da população estudos sobre essa temática devem ser estimulados. A importância de recorte étnico-racial relativo às doenças e condições de vida dos quilombolas permite que seja quantificado a parcela da população que está mais suscetível a desenvolvimento de doenças crônicas e agravos a saúde, a exemplo da hipertensão arterial e uma compreensão mais aprofundada acerca dessa relação pode facilitar medidas de promoção da saúde, prevenção de agravos, criação de tratamentos mais adequados e mudanças de comportamentos inadequados, proporcionando a essa parcela da população melhores condições de saúde 6.

Nesse contexto, devido a constituição histórica, a população afro-brasileira quilombola tem maior predominância de negros do que a população geral brasileira 7.

Este estudo tem como objetivo analisar o sobrepeso, obesidade e os fatores associados à saúde em afrodescendentes hipertensos residentes em uma comunidade quilombola e a questão norteadora foi: quais os fatores de risco cardiovascular estão associados a sobrepeso e obesidade em afrodescendentes hipertensos residentes em comunidade quilombola? A hipótese do estudo é: quanto maior a presença de sobrepeso e obesidade, maior a magnitude de associação com os fatores associados à saúde em afrodescendentes residentes em comunidade quilombola.

## MÉTODOS

### Desenho do estudo e população

Trata-se de um estudo epidemiológico, censitário, de delineamento transversal e de base comunitária, que faz parte do projeto intitulado 'Avaliação clínica do risco cardiovascular e fatores associados a adesão terapêutica de pessoas em famílias quilombolas".

A pesquisa foi realizada respeitando os aspectos éticos referentes à Resolução 466/2012, do Conselho Nacional de Saúde, relacionado à pesquisa envolvendo seres humanos e foi submetido ao Comitê de Ética em Pesquisa (CEP) pela Faculdade Independente do Nordeste - FAI-NOR sob CAAE: 56705617.2.0000.5578 e aprovado sob o parecer de número: 2.015.327 de 2017.

O estudo aconteceu no período de novembro de 2017 à março de 2018, na Comunidade Quilombola do Barro Preto, no município de Jequié-BA, a partir dos domicílios registrados nas áreas de abrangência da Unidade de Saúde da Família Odorico Motta, vinculada à Secretaria Municipal de Saúde e inserida na comunidade quilombola do Barro Preto, no município de Jequié-BA.

Foi realizado o levantamento epidemiológico nos arquivos do prontuário familiar da usf local, que resultaram em uma amostra de 400 pessoas para a coleta de dados. Entretanto, para este estudo, foram analisadas uma amostra de 303 pessoas afrodescendentes residentes em comunidade quilombola, que atenderam ao critério de elegibilidade.

Os critérios de elegibilidade para a seleção foram: -Pessoas remanescentes quilombolas residentes na comunidade do Barro Preto que se autodeclaram quilombolas; - Cadastradas na usf Odorico Mota; - De ambos os sexos, adultos e idosos; - Faixa etária de 35 a 79 anos; - Diagnóstico de Hipertensão Arterial Sistêmica; - Em uso de anti-hipertensivos.

Foram exclusos desse estudo: pessoas que foram identificadas no levantamento epidemiológico e não foram encontradas no momento da entrevista, sendo realizada três tentativas em dias e horários diferentes; que não estavam em condições clínicas de participar da pesquisa devido a comprometimento físico, mental ou emocional; que não possuíam resultados de exames laboratoriais atualizados (realizados há menos de seis meses) ou não compareceram a etapa de coleta sanguínea da pesquisa.

### Procedimentos

A coleta de dados foi realizada em duas etapas. Na primeira, foi realizada uma avaliação clínica, onde os participantes preencheram questionários, tiveram sua composição corporal avaliada e a pressão arterial medida durante a entrevista realizada em suas residências. A segunda etapa consistiu na coleta de sangue venoso, que foi feita na usf localizada na comunidade onde ocorreu o estudo. Os exames foram analisados no laboratório de referência central, conforme métodos automatizados específicos e padronizados.

Utilizou-se o Questionário de Hipertensão Arterial na Atenção Primária à Saúde, para investigar informações relacionadas à saúde dos participantes, como aspectos clínicos, epidemiológicos, terapêuticos e dados sociodemográficos. Também foram avaliados fatores de risco, comorbidades e o acesso ao diagnóstico, tratamento e serviços de saúde, bem como, a mensuração da pressão arterial e mensuração antropométrica da composição corporal 8.

Na avaliação clínica da composição corporal, foram aferidos os parâmetros de peso, altura, IMC e circunferência da cintura, seguindo as instruções do manual de antropometria da Pesquisa Nacional de Saúde (PNS) 9. A balança de bioimpedância digital e o estadiômetro portátil fo ram utilizados para obter medidas precisas da composição corporal. A circunferência da cintura foi medida com uma fita métrica e foram adotados pontos de corte específicos. Esses procedimentos foram muito importantes para a obtenção de medidas precisas e confiáveis da composição corporal dos participantes.

A circunferência da cintura (CC) foi medida em centímetros (cm) utilizando uma fita métrica. Os pontos de corte adotados foram CC ≥ 102 cm para homens e CC ≥ 88 cm para mulheres, seguindo os critérios estabelecidos pelo *National Cholesterol Education Program III* (NCEP III) 10.

O IMC foi calculado dividindo-se a massa corporal pelo quadrado da estatura. Os participantes foram classificados como tendo excesso de peso ou eutróficos, de acordo com os critérios da Organização Mundial da Saúde. 11.

A pressão arterial foi medida com um esfigmomanômetro digital e registrados os valores da pressão arterial sistólica e diastólica, seguindo as diretrizes brasileiras de hipertensão. 12.

### Análise estatística

Realizou-se através de testes da estatística descritiva e inferencial. Os resultados foram analisados em percentuais e médias e apresentados em forma de figura e tabelas para melhor visualização dos achados, e discutidos com base na literatura atual nacional e internacional acerca do tema.

Os dados foram analisados usando o software Statistical Package for Social Sciences - SPSS for Windows, versão 21.0, com um nível de significância de p < 0,05. Com o objetivo de investigar as variáveis que influenciam a ocorrência de obesidade em comunidades quilombolas e estimar a magnitude dessa influência, foram utilizados diversos modelos de regressão logística binária via GLM (Generalized Linear Model). Esses modelos foram empregados para analisar a relação entre as variáveis quantitativas explicativas e o desfecho obesidade, categorizado IMC > 25 (Com sobrepeso e obesidade -Sem sobrepeso ou obesidade).

## RESULTADOS

Uma amostra de 303 pessoas foi analisada, desta obteve-se os seguintes resultados: 71,3% (n=216) eram do sexo feminino e 28,7% (n= 87) eram do sexo masculino. A idade média dos participantes foi de 59,8 ± 11,3 anos. 22,4% (n=68) dos participantes não apresentavam sobrepeso, enquanto a maioria (76%, ou n=231 pessoas) apresentava sobrepeso. A maioria morava com familiares sem companheiro (31,5%, n= 95), 28,4% (n=86) moravam com companheiro e filho, 21,5% (n=65) com companheiro sem filho, 9,2% (n=28) viviam sozinhos, 6,9% (n=21) com companheiro/filhos/outros e 2,3% (n=7) com companheiro/não parentes/sem laços. Quanto à escolaridade dos participantes, 29% (n=88) eram analfabetos, 12,5% (n=38) eram alfabetizados, 39,3% (n=119) tinham ensino fundamental incompleto, 8,6% (n=26) tinham ensino fundamental completo, 4% (n=12) tinham ensino médio incompleto, 5,9% (n=18) tinham ensino médio completo e 0,7% (n=2) tinham ensino superior completo.

A renda média dos participantes foi de 1089 ± 476 reais. A maioria era da raça/cor preta (49%, ou 149 pessoas), parda (41,3%, ou 125 pessoas), branca (7,9%, ou 24 pessoas), amarela (1,3%) e indígena (0,3%). Além disso, 55,6% (169 pessoas) disseram ter antecedentes familiares de doença cardiovascular. A maioria das participantes apresentava menopausa (58,1%), antecedentes familiares cardiovasculares (55,8%), sedentarismo (50,5%), diabetes tipo 2 (23,4%), etilismo (16,6%), tabagismo (9,2%), outras coronariopatias (8,6%), doença renal (6,6%), uso de contraceptivo hormonal (2,6%), infarto agudo do miocárdio (2,3%), pé diabético (0,3%) e amputação por diabetes (0,3%) (Tabela 1).

**Tabela 1:** Fatores de risco cardiovascular em afrodescendentes hipertensos. Jequié, Bahia, Brasil, 2023

Menopausa	176	58,1
Antecedente familiar cardiovascular	169	55,8
Sedentarismo	153	50,5
Diabetes T2	71	23,4
Etilismo	50	16,6
Tabagismo	28	9,2
Outra coronariopatias	26	8,6
Doença renal	20	6,6
AVC	16	5,3
Diabetes T1	9	3,0
Uso de contraceptivo hormonal	8	2,6
Infarto agudo miocárdio	7	2,3
Pé diabético	1	0,3
Amputação por diabetes	1	0,3

Fonte: Elaborado pelos autores, 2023.

A média de peso das mulheres foi 69,1 ± 14,6 kg, altura média de 1,52 ± 0,07 m, cintura média de 100,9 ± 17,5 cm e idade média de 59,4 ± 11,4 anos. No grupo dos homens, a média de peso foi 72,7 ± 16,2 kg, altura média de 1,62 ± 0,07 m, cintura média de 96,6 ± 19,2 cm e idade média de 60,7 ± 11,1 anos.

Nas mulheres, o perfil lipídico: a média dos triglicerídeos foi de 209,6 ± 236,1 mg/dl, o colesterol total foi de 215,9 ± 49,7 mg/dl, o colesterol HDL foi de 37,4 ± 10,4 mg/dl, o colesterol LDL foi de 140,4 ± 40,8 mg/dl e a glicemia em jejum foi de 112,9 ± 58,9 mg/dl. Nos homens, a média dos triglicerídeos foi de 172,3 ± 90,5 mg/dl, o colesterol total foi de 195,5 ± 40,9 mg/dl, o colesterol HDL foi de 32,3 ± 11,8 mg/dl, o colesterol LDL foi de 127,6 ± 35,9 mg/dl e a glicemia em jejum foi de 102,2 ± 40,7 mg/dl (tabela 2). Foram encontradas diferenças estatisticamente significativas no colesterol total (p<0,001), colesterol HDL (p<0,000) e colesterol LDL (p<0,016) das mulheres quando comparados com a média dos homens.

**Tabela 2:** Perfil lipídico segundo o sexo em afrodescendentes hipertensos. Jequié, Bahia, Brasil, 2023.

	Femenino (n=133)		Masculino (n=54)		
	Média ± D.P		Média ± D.P		p valor*
Triglicéridos	209,7	236,2	172,3	90,5	0,725
Colesterol Total	215,9	49,7	195,6	40,9	0,001
HDL	37,4	10,4	32,3	11,9	0,000
LDL	140,4	40,9	127,6	36,0	0,016
Glicemia jejum	112,9	58,9	102,2	40,8	0,213

Teste não paramétrico Mann-Whitney*

No modelo multivariado, após o ajuste para renda e glicemia, diversas variáveis mostraram-se estatisticamente significantes em relação à obesidade. O sexo (p<0,004), o colesterol HDL (p<0,005), o colesterol total (p<0,01), o colesterol LDL (p<0,01) e a idade (p<0,0001) apresentaram associação estatisticamente significante com o desfecho.

As mulheres quilombolas apresentaram 2,6 vezes mais chances de desenvolver obesidade em comparação aos homens (OR: 2,6; IC95%: 1,3-5,2). O colesterol HDL demonstrou um efeito protetor significativo (OR: 0,95; IC95%: 0,920,98), assim como a idade (OR: 0,94; IC95%: 0,91-0,97). Por outro lado, o colesterol total mostrou-se um fator de risco para obesidade (OR: 1,02; IC95%: 1,007-1,05) (Tabela 3).

**Tabela 3:** Ajuste multivariado para os fatores associados a obesidade em quilombolas. Jequié, Bahia, Brasil, 2023

Parâmetro	OR	Inferior	Superior	p value
Sexo	2,69	1,36	5,28	0,004*
HDL	0,95	0,92	0,98	0,005*
Colesterol Total	1,02	1,00	1,05	0,011*
Idade	0,94	0,91	0,97	0,0001*
LDL	0,97	0,94	0,99	0,011*
Glicemia jejum	1,00	0,99	1,01	0,107
Renda	1,00	0,99	1,00	0,408

^*^ Associações estatisticamente significantes.

## DISCUSSÃO

A partir dos dados apresentados foi possível observar que a maioria dos participantes apresentava sobrepeso (76%, ou 231 pessoas) e que a idade média em relação a idade era de 59,8 ± 11,3 anos. Esses dois fatores são importantes indicadores de risco para doenças cardiovasculares, especialmente quando combinados.

A idade é um dos principais fatores de risco para doenças cardiovasculares devido às alterações fisiológicas que ocorrem no sistema cardiovascular à medida que as pessoas envelhecem, e aponta que as principais mudanças fisiológicas que acometem os idosos acarretando problemas coronários são: Espessamento das artérias, Diminuição da elasticidade das artérias e Disfunção diastólica, facilitando assim uma diminuição da função cardíaca 13.

A baixa testosterona está associada a obesidade e aponta também que há uma relação inversa entre a Testosterona e o IMC, pois, a obesidade e a baixa testosterona têm uma relação de mão dupla, em que a obesidade pode levar ao decréscimo do nível desse hormônio, que por sua vez pode agravar a obesidade. Esse fator é importante principalmente em homens idosos, devido a diminuição fisiológica do nível desse hormônio, que pode facilitar assim o desenvolvimento de sobrepeso e obesidade em homens com mais de 30 anos 14.

Todas essas mudanças têm implicações para doenças cardiovasculares, como doenças coronarianas e a hipertensão. Que muitas vezes está associado a um estilo de vida sedentário, frequentemente encontrado em pessoas com mais de 65 anos de idade. Além disso, o corpo humano tende a ter uma resposta inflamatória menos efetiva com o passar do tempo, o que pode afetar a capacidade do corpo de se recuperar totalmente após uma lesão, como acontece nos casos de IAM (infarto agudo do miocárdio) e até mesmo no AVE (acidente vascular encefálico) 15.

Podemos observar que o número de mulheres na menopausa foi consideravelmente alto (58,1% da amostra n=176). É sabido que a menopausa é um fator de risco para o desenvolvimento do excesso de peso por vários motivos como alterações na composição corporal, diminuição da massa muscular, redução da taxa metabólica basal e também as alterações hormonais, que afetam principalmente os níveis de estrogênio, a diminuição do nível desse hormônio é muito importante, pois pode levar a alterações no metabolismo da mulher, gerar alterações no perfil lipídico e alterar a redistribuição da gordura corporal facilitando o depósito de gordura na região abdominal. Sendo que a gordura abdominal está associada a um maior risco de desenvolvimento de doenças cardiovasculares 16.

Dessa forma foi observado também que as mulheres estão em maior risco de desenvolver o sobrepeso do que os homens, visto que a menopausa em si é um fator importante para o desenvolvimento do sobrepeso. Além do sobrepeso, as mulheres são mais propensas a se tornarem obesas por uma variedade de razões, incluindo variáveis biológicas, hormonais, comportamentais e sociais 16.

Na amostra pode-se observar que 23,4% (n = 71) das pessoas entrevistadas são portadores de Diabetes Mellitus, o que é um dado preocupante, pois a diabetes é fator de risco para o desenvolvimento de vários tipos de condições e complicações, como as doenças cardiovasculares 17.

Em um estudo realizado em 2022 afirmou-se que a obesidade, juntamente com o sobrepeso, está associada as várias alterações metabólicas que podem aumentar o risco de desenvolver doenças crônicas. Uma dessas alterações, associadas à obesidade, é a resistência à insulina que ocorre quando as células do corpo não respondem adequadamente à insulina podendo causar o quadro da diabetes tipo 2, evidenciado que existe uma ligação direta entre alterações na glicemia e o ganho de peso 17.

A obesidade é um fator de risco importante para as doenças cardiovasculares por vários motivos. Primeiro, o excesso de gordura corporal pode aumentar os níveis de colesterol, LDL e triglicerídeos no sangue, o que pode levar à formação de placas de gordura nas paredes das artérias (aterosclerose). A obesidade também pode influenciar nos baixos níveis de HDL que é importante para o funcionamento do organismo 18.

Neste estudo foi observado que a taxa de HDL foi de 37,4 para as mulheres e 32,3 mg/dl para os homens, o que é uma taxa preocupante, pois o que se espera para um HDL em nível considerado normal é de 40 e 50mg/dl (Mulheres e Homens respetivamente). Um estudo em 2023 afirmou que o HDL é conhecido como "colesterol bom", pois altos níveis de HDL-C estão associados a um menor risco de doenças cardiovasculares. Isso ocorre porque o HDL tem muitas funções benéficas para o organismo, incluindo a remoção do excesso de colesterol das artérias, a inibição da oxidação do LDL-C (conhecido como "colesterol ruim") e a redução da inflamação nas paredes arteriais. Em resumo, o HDL ajuda a manter as artérias saudáveis, protegendo contra doenças cardiovasculares 18.

A obesidade é um dos principais fatores de risco para o desenvolvimento de doenças crônicas e cardíacas, como o diabetes mellitus tipo 2 e a doença arterial coronariana. A obesidade está associada a uma série de alterações metabólicas e fisiológicas que podem levar ao desenvolvimento dessas doenças, incluindo o aumento dos níveis de colesterol e triglicerídeos no sangue, a resistência à insulina, a inflamação crônica de baixo grau e a disfunção endotelial 13.

A alta porcentagem de participantes com sobrepeso e sedentarismo (50,5% ou 153 pessoas) sugere um risco aumentado para problemas cardiovasculares, como infarto do miocárdio, acidente vascular cerebral, doença arterial coronariana, entre outros 19.

A obesidade e o sedentarismo são fatores de risco bastante conhecidos para doenças cardiovasculares, e a presença de antecedentes familiares aumenta ainda mais esse risco. Além disso, a obesidade está frequentemente associada a outros fatores de risco cardiovascular, como a hipertensão arterial, resistência à insulina e inflamação crônica. Esses fatores podem ser controlados justamente com um estilo de vida saudável e ativo, quando o estilo de vida sedentário é a realidade, pode ocorrer agravos desses fatores de risco e ocasionar diversos quadros clínicos preocupantes como: levar ao estreitamento das artérias e à redução do fluxo sanguíneo para o coração e outros órgãos importantes 19.

A renda média dos participantes do estudo foi de 1089 ± 476 reais, esse valor pode ter um impacto significativo na qualidade de vida dessas pessoas e no risco de sobrepeso e obesidade, por ser um valor consideravelmente baixo. Embora a renda não seja o único fator que influencia a saúde, ela pode desempenhar um papel fundamental na determinação do acesso a alimentos saudáveis, atividades físicas e acessos aos serviços de saúde adequados 20.

O sobrepeso e a obesidade são influenciados pela renda dos indivíduos de maneiras diversas. Na maioria das vezes as pessoas de baixa renda têm maior probabilidade de apresentar sobrepeso ou obesidade do que pessoas de alta renda. Algumas das razões incluem: alimentação inadequada, falta de atividade física, dificuldade financeira, acesso limitado à informação e educação em saúde, fatores culturais e sociais e muitos outros fatores 21.

Em uma pesquisa de revisão de estudos epidemiológicos encontrou evidências de que indivíduos que vivem em bairros com baixo status socioeconômico têm maior probabilidade de apresentar sobrepeso ou obesidade em comparação com aqueles que vivem em bairros com alto status socioeconômico 20.

Os povos quilombolas enfrentam desafios significativos em relação ao sobrepeso e à obesidade, e entender esses fatores que influenciam nessa realidade é muito importante para o desenvolvimento de abordagens eficazes de prevenção e intervenção. Além disso, esses povos, muitas vezes, enfrentam desafios socioeconômicos e de acesso aos serviços de saúde, o que pode dificultar a prevenção e o tratamento do sobrepeso e da obesidade 6.

Um dos principais fatores contribuintes foram a mudança no estilo de vida e nos hábitos alimentares. Com a modernização, muitos quilombolas passam por uma transição nutricional, abandonando sua dieta tradicional baseada em alimentos naturais e adotando uma alimentação ocidentalizada, caracterizada por alimentos processados, açúcares e gorduras. Essa mudança resulta em um aumento no consumo de calorias vazias e nutrientes inadequados, o que contribui para o ganho de peso 22.

Esses fatores de risco estão ligados diretamente às desigualdades socioeconômicas que os povos quilombolas enfrentam. A pobreza, a falta de oportunidades educacionais, o desemprego e o acesso limitado aos serviços de saúde são realidades comuns nessas comunidades. Esses fatores socioeconômicos têm um impacto significativo nas escolhas alimentares e no estilo de vida dessa parcela da população tornando assim, mais difícil a adoção de hábitos e escolhas saudáveis 20.

Além disso, o acesso limitado a alimentos saudáveis é outro fator desafiador. Muitas comunidades quilombolas enfrentam dificuldades relacionadas à segurança alimentar, com acesso restrito a alimentos frescos, nutritivos e culturalmente adequados. A falta de infraestrutura e a distância dos centros urbanos dificultam a obtenção de alimentos saudáveis, levando a escolhas menos benéficas para a saúde. O contexto histórico e cultural não pode ser ignorado. Os povos quilombolas carregam consigo uma herança de discriminação, marginalização e acesso limitado a recursos. Esses fatores históricos e estruturais têm um impacto profundo na saúde dessas comunidades, afetando negativamente seus hábitos alimentares, estilos de vida e acesso a cuidados de saúde adequados 22.

As limitações com relação a este estudo foram: delineamento transversal, com participação voluntária restrita de afrodescendentes de uma única comunidade quilombola urbana. Portanto, fica limitado terce generalizações para população brasileira. As informações nutricionais das participantes não foram investigadas. Os resultados sugerem que estudos longitudinais possam investigar os feitos do sobrepeso/ obesidade que incidem sobre os fatores associados à saúde cardiovascular em afrodescendentes residentes em comunidade quilombola.

A partir desse estudo pode-se observar que os povos quilombolas estão relativamente propensos ao desenvolvimento de doenças crônicas não transmissíveis, em especial as doenças cardiovasculares, bem como evidenciou-se na amostra analisada características socioeconômicas e de saúde muito preocupantes. A maioria dos participantes era do sexo feminino, apresentava sobrepeso, baixa escolaridade, baixa renda e relatou ter antecedentes familiares de doença cardiovascular e fatores cardiodeletérios, como menopausa, sedentarismo, diabetes tipo 2, etilismo e tabagismo.

Esses achados são importantes pois indicam uma alta prevalência de fatores de risco para doenças cardiovasculares nos povos quilombolas, implicando em graves problemas de saúde e causando impactos negativos na saúde e qualidade de vida dessa população. A obesidade e o sobrepeso se destacaram dentre os principais fatores de risco, associados também à idade, sexo, sedentarismo, baixa renda e alterações metabólicas.

A relação entre excesso de peso e idade é importante, pois o envelhecimento está associado a alterações fisiológicas no sistema cardiovascular, como espessamento arterial, perda de elasticidade e disfunção diastólica, que podem levar a problemas nas artérias coronárias. Além disso, a baixa testosterona em homens mais velhos pode contribuir para o desenvolvimento de sobrepeso e obesidade.

Em relação às alterações metabólicas, a presença de diabetes nas amostras indicou uma relação direta entre ganho de peso e hiperglicemia. A obesidade também está associada a outras condições metabólicas, como pressão alta, devido a níveis elevados de colesterol, triglicerídeos e efeitos da inflamação crônica.

O baixo HDL também se mostrou como um fator de risco preocupante na parcela em questão, visto que a média dos níveis de HDL estava abaixo do valor ideal, podendo ocasionar complicações e danos à saúde, como: doenças cardíacas coronárias, aterosclerose, hipertensão entre outras.

O sedentarismo, juntamente com a baixa renda, também se mostrou como fatores de risco alarmantes devido sua alta prevalência. A baixa renda pode afetar o acesso a alimentos saudáveis, atividades físicas e serviços de saúde adequados e, como um pouco mais da metade dos participantes relataram ter um estilo de vida sedentário, combinado com o sobrepeso e a obesidade, esses fatores aumentam ainda mais o risco de problemas cardiovasculares, como infarto agudo do miocárdio, acidente vascular cerebral e doença arterial coronariana. O estudo evidenciou que indivíduos de baixa renda e com estilo de vida sedentário têm maiores chances de apresentar sobrepeso e obesidade devido diversos fatores.

Ao considerarmos a população quilombola deparamo-nos com desafios de magnitude ampliada. A transição nutricional, a dificuldade de acesso a alimentos saudáveis, as desigualdades socioeconômicas enfrentadas, como a pobreza, a carência de oportunidades educacionais, desemprego e a limitação no acesso aos serviços de saúde, são dificuldades adicionais na prevenção, tratamento e controle do sobrepeso e da obesidade, contribuindo assim para uma crescente prevalência de sobrepeso e obesidade nessas comunidades.

Em síntese, os resultados ressaltaram a necessidade da criação de estratégias preventivas e intervencionistas eficazes no combate ao sobrepeso e à obesidade, em especial nas populações vulneráveis. É imprescindível estimular a educação em saúde, aprimorar o acesso a alimentos saudáveis, incentivar a prática regular de atividade física e combater as disparidades socioeconômicas, visando a mitigar os impactos desses fatores de risco e aprimorar a saúde cardiovascular da população em estudo.

Este estudo se mostra relevante e pode ser utilizado como ferramenta de análise importante para que futuras medidas de intervenções multiprofissionais, sociais e de educação em saúde baseada nos agravos de saúde mais prevalente na comunidade, na perspectiva de tomar decisões assertivas com relação a população da comunidade quilom-bola, bem como em outras comunidades vulneráveis ♥
